# Time-Resolved cAMP Level Determination in Frog Retina Samples Using LC–MS/MS

**DOI:** 10.21769/BioProtoc.5431

**Published:** 2025-09-05

**Authors:** Olga V. Chernyshkova, Mikhail V. Belyakov, Darya A. Meshalkina, Mikhail L. Firsov

**Affiliations:** 1 Sechenov Institute of Evolutional Physiology and Biochemistry, RAS, St. Petersburg, Russia; 2 Research Institute of Hygiene, Occupational Pathology and Human Ecology of the Federal Medical-Biological Agency, St. Petersburg, Russia

**Keywords:** Retina, cAMP, LC–MS/MS, Photoreceptor, Phototransduction

## Abstract

The phototransduction cascade allows photoreceptors to detect light across a wide range of intensities without saturation, with cGMP serving as the second messenger and calcium feedback as the key regulatory mechanism. While experimental evidence suggests that cAMP may also play a role in modulating this cascade, such regulation would necessitate rapid changes in cAMP levels on a timescale of seconds. However, data on the dynamics of intracellular cAMP changes in photoreceptors remain scarce, primarily due to the limitations of conventional fluorescence-based methods in this specialized sensory system. To address this gap, we developed a methodology combining rapid cryofixation of retinal samples following light stimulation with the isolation of outer segment preparations. The rapid cryofixation setup comprises six computer-controlled sections, each with a high-speed stepper motor-driven lever that rapidly moves the specimen in a 180° arc within ~80 ms to press it against a liquid nitrogen-cooled copper cylinder for fixation. Using highly sensitive metabolomics techniques, we measured cAMP levels in these samples. This approach enables the investigation of rapid cAMP dynamics and its potential regulatory role in phototransduction, providing a foundation for understanding the interplay between cAMP and PKA signaling in photoreceptor function.

Key features

• The protocol provides ms time resolution in retina outer segment sampling in response to light stimulus with cryofixation, conserving proteome and metabolome response features.

• The protocol allows direct cAMP quantification with an average level of 11.4 ± 0.5 pmol/mg of protein in the dark.

## Graphical overview


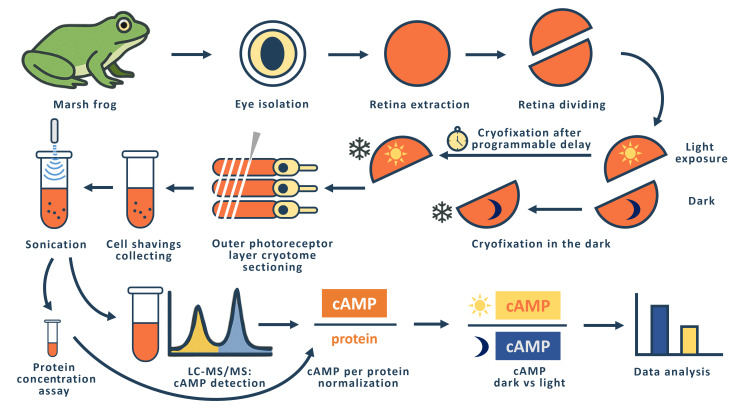


## Background

In the realm of vertebrate visual systems, the phototransduction cascade plays a crucial role in enabling effective vision across a vast spectrum of light intensities, from the dimmest of night conditions to the brightness of midday. This intricate biochemical pathway operates through a multi-stage amplification system, characterized by several feedback loops, allowing photoreceptors to adapt to varying illumination levels. The secondary messengers involved in this process, cyclic guanosine monophosphate (cGMP) and cyclic adenosine monophosphate (cAMP), each serve distinct yet interrelated functions within the cascade. While the role of cGMP in regulating the permeability of photoreceptor plasma membrane channels has been extensively documented [1,2], the nuanced roles of cAMP have garnered less attention, despite its significant involvement in various photoreceptor processes, including modulation of circadian rhythms and the dynamics of retinomotor movements [3–8].

Previous studies have demonstrated that several key proteins within the phototransduction pathway—such as phosphodiesterase type 6 (PDE6) and rhodopsin kinase—are susceptible to phosphorylation by protein kinase A (PKA), suggesting a regulatory interplay between cAMP levels and the efficiency of the phototransduction cascade [8,9]. However, the dynamics of cAMP concentration within photoreceptors remain inadequately characterized, particularly concerning the rapid fluctuations necessary for real-time regulation of the cascade's activity.

Existing protocols for measuring intracellular signaling, including cAMP dynamics, have predominantly relied on fluorescent methods, which are often unsuitable for the unique conditions pertinent to photoreceptor physiology. The potent light emission employed in the fluorescence technique itself constitutes an adequate stimulus for the phototransduction cascade. The rapid cryofixation technique utilized in this method presents significant advantages over these conventional approaches, allowing for precise measurement of cAMP levels immediately following light stimulation. By employing highly sensitive metabolomics techniques paired with cryofixed samples, we provide a robust protocol that significantly enhances our ability to capture real-time biochemical changes during phototransduction.

The general idea of rapid freezing of retinal fragments was previously proposed and realized by Govardovskii [10] and Blazynski and Cohen [11, 12], who used this approach to study the effect of light stimulation on intracellular cGMP levels in amphibian outer segments. In both cases, the cryofixation surface was a mirror-polished surface of a copper cylinder cooled to the temperature of liquid nitrogen [10] or liquid helium [11]. The Govardovskii setup allowed simultaneous fixation of four retinal fragments with a minimum sample cryofixation time of 500 ms. We refer to the minimum time here as the time interval from command to cryofixation of the specimen. The Blazinski setup was a single-channel unit with a minimum cryofixation time of 47 ms.

In designing our setup, we considered that it should be multi-channel and allow us to fix retinal fragments from two eyes of an animal in one experiment. Experimentally, we have found that the optimal size of a retinal fragment for obtaining a tissue sample using a cryotome is 1/2 to 1/3 of the retina of one eye. Thus, in one experiment, it is possible to simultaneously fixate and then measure cAMP levels in 4–6 retinal fragments from two eyes of an animal. To perform such an experiment, we created a setup consisting of six identical sections in which a fast-moving stepper motor (maximum speed 150,000 rpm) drives a lever with a retinal sample pad. The retinal sample, placed on a small circle of filter paper, is placed horizontally on the pad and is illuminated with LED light (λ_max_= 525 nm) on command from the computer. The intensity of the light used to stimulate the retina can be varied within a range of 3.6 × 10^3^–3.6 × 10^5 ^quanta/(s·μm^2^). If necessary, a grey light filter can be used to further attenuate the light flux. Synchronous illumination of all six retinal specimens is achieved by a special design of the light guide that carries the light from the LED to the retinal specimens. The light guide has six identical fiber bundles at one end, each of which illuminates one location with a retinal fragment. At the other end, the fibers from all six terminations are combined into a single bundle by uniform and random mixing. The end of this bundle is illuminated through a condenser by an LED.

After a programmable delay, the sample arm arcs 180° and presses the retinal sample against the polished surface of a copper cylinder cooled to liquid nitrogen temperature. The time taken for the lever to move from the command stage to making contact with the surface of the copper cylinder (the minimum sample cryofixation time) was measured using a slow-motion video of the lever's rotation. The results of multiple measurements showed a small variation in the individual minimum cryofixation times, which for channels 1–6 are 153 ± 12, 94 ± 9, 92 ± 3, 97 ± 6, 90 ± 1, and 110 ± 2 (shown as average ± SD, ms, n = 3–5). The average time for all channels is 106 ms. We used the measured minimum cryofixation times to calculate the actual cryofixation delay time for each individual channel. To prevent the retinal specimens from flying off the platform during the rapid movement of the lever, we glued filter paper circles on which the retinal specimens were placed to the platform on the lever using Tissue-Tek gel. To enable the Tissue-Tek frozen after cryofixation to be easily removed from the lever platform, we made a composite platform with a fluoroplastic core.

## Materials and reagents

**Biological materials**

Adult marsh frogs (*Pelophylax ridibundus*) were collected from the wild in southern Russia. Frogs were housed in water tanks at 6–8 °C for a maximum of 8 months. Animals were handled in accordance with the Council Directive of the European Communities (24 November 1986; 86/609/EEC), and the experimental protocol was approved by the local Institutional Animal Care and Use Committee (protocol # 4/22 at 28.01.2022).

**Reagents**

1. Liquid nitrogen

2. Sodium chloride (NaCl) (Sigma-Aldrich, catalog number: S7653)

3. Potassium chloride (KCl) (Sigma-Aldrich, catalog number: P9333)

4. Magnesium chloride (MgCl_2_) (Sigma-Aldrich, catalog number: M1028)

5. Calcium chloride (CaCl_2_) (Sigma-Aldrich, catalog number: 21115)

6. Sodium bicarbonate (NaHCO_3_) (Sigma-Aldrich, catalog number: S5761)

7. HEPES (Sigma-Aldrich, catalog number: H3375)

8. Glucose (D-Glucose) (Sigma-Aldrich, catalog number: G8270)

9. Ethylenediaminetetraacetic acid (EDTA) (Sigma-Aldrich, catalog number: E5134)

10. Sodium hydroxide (NaOH) (Sigma-Aldrich, catalog number: 72068)

11. Tissue-Tek (Sakura, catalog number: 4583)

12. Coomassie Brilliant Blue G-250 (Helicon, catalog number: SRL-64222-5G)

13. Ethanol (ethyl alcohol, 96%) (LenReaktiv, catalog number: 000164)

14. Phosphoric acid (H_3_PO_4_) (Sigma-Aldrich, catalog number: 345245)

15. Bovine serum albumin (BSA) (Dia-M, catalog number: BSA.0100)

16. Cyclic adenosine monophosphate (cAMP) (Merck, catalog number: A9501)

17. Сyclic guanosine monophosphate (cGMP) (Merck, catalog number: G6129)

18. Acetonitrile (Cryochrom, Russia)

19. Hydrochloric acid (Supelco, catalog number:109057)

20. Ammonium formate (CDH, catalog number: 027169)

21. Isotopic-labeled cyclic adenosine monophosphate (cAMP-13C5) (TRC, Canada, catalog number: A280457)

**Solutions**

1. Frog Ringer (FR) (see Recipes)

2. Bradford reagent (see Recipes)

3. Mass-spec solution (see Recipes)

**Recipes**

**1. Frog Ringer (FR)**

**Table:** 

Reagent	Final concentration	Quantity or Volume
NaCl	90 mM	18 mL, 5 M
KCl	2.5 mM	2.5 mL, 1 M
MgCl_2_	1.4 mM	1.4 mL, 1 M
CaCl_2_	1.05 mM	1.05 mL, 1 M
NaHCO_3_	5 mM	5 mL, 1 M
HEPES	5 mM	5 mL, 1 M
Glucose	10 mM	10 mL, 1 M
EDTA	0.05 mM	0.1 mL, 0.5 M
NaOH	To pH 7.6	
MQ water	To 1,000 mL	

**2. Bradford reagent**

**Table:** 

Reagent	Final concentration	Quantity or Volume
Ethanol	5%	50 mL
Coomassie G-250	100 mg/L	100 mg
H_3_PO_4_	10%	100 mL
MQ water	To 1,000 mL	

**3. Mass-spec solution**

**Table:** 

Reagent	Final concentration	Quantity or Volume
Ammonium formate	0.1 M	6.30 g
MQ water	To 1,000 mL	

**Laboratory supplies**

1. Microcentrifuge tubes, 0.5 and 1.5 mL (SSIbio, catalog numbers: 1110-00 and 1210-00)

2. Filter paper (ECOS-1, catalog number: A200042894902)

3. 96-well (Medpolymer, catalog number: 112202) and 6-well plates (NEST, catalog number: 703011)

4. 3 cm (Jet Biofil, catalog number: TCD000035) and 10 cm Petri dishes (Medpolymer, catalog number: 124124)

5. Hollow metal tube: custom-made copper tube with an outer diameter of 8 mm and a wall thickness of 0.7 mm

6. Metal spatulas

7. Dissecting scalpels and blades, dissecting scissors, dissecting dressing tweezers

8. Light-isolating cryo-container: custom-made, constructed from a metal box filled with liquid nitrogen, featuring metal cylinders that support the sample platform above; the exterior of the cryocontainer is insulated with foam to minimize thermal exchange

## Equipment

1. Centrifuge for Eppendorf tubes (Eppendorf miniSpin plus, Eppendorf, Germany)

2. Sonicator (Elmasonic S30, Elma-Hans Schmidbauer GmbH, Germany)

3. Vortex MultiReax (Heidolph instruments GmbH, Germany)

4. Chromatography column (Agilent Zorbax SB-C8 150 mm × 4.6 mm × 1.8 μm, Agilent Technologies, USA)

5. High-performance liquid chromatograph Dionex UltiMate 3000 with QExactive high-resolution mass-spectrometry detector with electrospray ionization (Thermo Fisher Scientific)

6. AUW-220D analytic balance (Shimadzu, Japan)

7. Hole punch with hole diameter of 1 cm

8. Red light/dark room

9. Infrared observation system (custom-made)

10. Milli-Q^®^ water system (Merck Life Science, model: Ultrapure, Type 1)

11. Stereomicroscope (Nexcope, model: NSZ-810)

12. Cryotome (Leica, rotary freezing cryotome, model: RM2265)

13. Sonicator (Sonics, model: Vibra-Cell, VCX130)

14. Drybath (Hangzhou Allsheng Instruments, model: MK-20)

15. CLARIOstar Plus microplate reader (Labtech International)

16. Cryofixation device (custom-made)

17. Lyophilizer (Labconco, catalog number: 7310031)

## Software and datasets

1. ThermoXcalibur Version 4.1.31.9 (Thermo Fisher Scientific)

2. Microsoft Excel 2010 (Microsoft)

## Procedure

**A. Preparation of retinal samples for cryofixation**

*Note: Operations from steps A2 to B6 must be performed at room temperature in a dark room under dim red light or using an infrared observation system to prevent rhodopsin bleaching.*

1. Preparations

a. Acclimatize animals to room temperature and a 12/12 h light cycle for several days prior to the experiment.

b. Dark-adapt the animals overnight.

c. Using a hole punch, punch 1 cm diameter discs from filter paper. Ensure that the discs remain clean by handling them with gloves and tweezers. Store the paper discs in a Petri dish.

**Figure 1.**
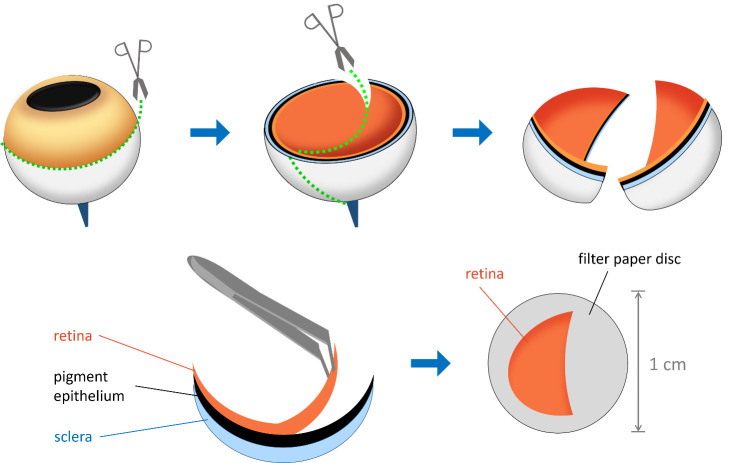
Schematic representation of retina isolation and layering. The eye is first sectioned along the indicated lines, and the two hemispheres are separated. The retina is then carefully detached from the pigment epithelium using tweezers. A sample of the isolated retina is layered with the photoreceptor side facing upward onto a 1 cm diameter filter paper disc.

2. Retina extraction

a. Decapitate the animal with scissors. After decapitation, destroy the brainstem using a thick needle.

b. Remove extraocular tissues with scissors.

c. Make an incision in the isolated eye with a scalpel on one side of the eyeball. Use spring scissors to cut along the equatorial line to hemisect the eye. Carefully remove and discard the anterior chamber, lens, and vitreous humor.

d. Repeat for the second eye. While the first retina is being processed, store the second eye semi-cup containing the neural retina in FR in a light-tight container at 4 °C until use.

e. Pour several milliliters of FR solution into a 3 cm Petri dish. Place the first eye semi-cup containing the neural retina into the solution, making sure it is completely submerged. Use scissors to cut the eye semi-cup into two, three, or four equal segments, depending on the experimental design (Figure 1).

f. Using a stereomicroscope, carefully separate the retina of each segment from the pigment epithelium with forceps. Discard the eye semi-cup segments containing the pigment epithelium. If any pigment epithelium remains on the retina, gently shake the retinal segment in the solution and then remove the remaining epithelium with tweezers, taking care not to damage the retina (Figure 1).

3. Layering the retina onto the filter paper disc

a. Using tweezers, place a filter paper disc into the same Petri dish.

b. Position the prepared retina with the photoreceptor side facing up on the filter paper disc. The photoreceptor side will be more concave inward, as it is in the eye in vivo (Figure 1).

c. Ensure that the retina is gently flattened on the filter paper. Use tweezers to carefully adjust the position.

d. Lift the filter paper disc out of the solution. As it emerges from the liquid, the retina will more easily straighten and spread across the filter paper.

e. Place the disc with the retinal segment on dry filter paper to remove excess liquid and enhance the adhesion of the retina to the paper disc.

*Note: This step is crucial, as failure to remove excess fluid may result in poor adhesion and detachment of the retinal segment. On the other hand, excessive drying may cause the segment to become brittle, break, and fall off the paper.*

f. Pour 3 mL of FR solution into each well of the 6-well plate. Place the sample in one of the wells and proceed with the preparation of the remaining samples from the same eye. Store the samples at room temperature in the dark for no more than 10 min before cryofixation.

**B. Light exposure and cryofixation**

**Figure 2.**
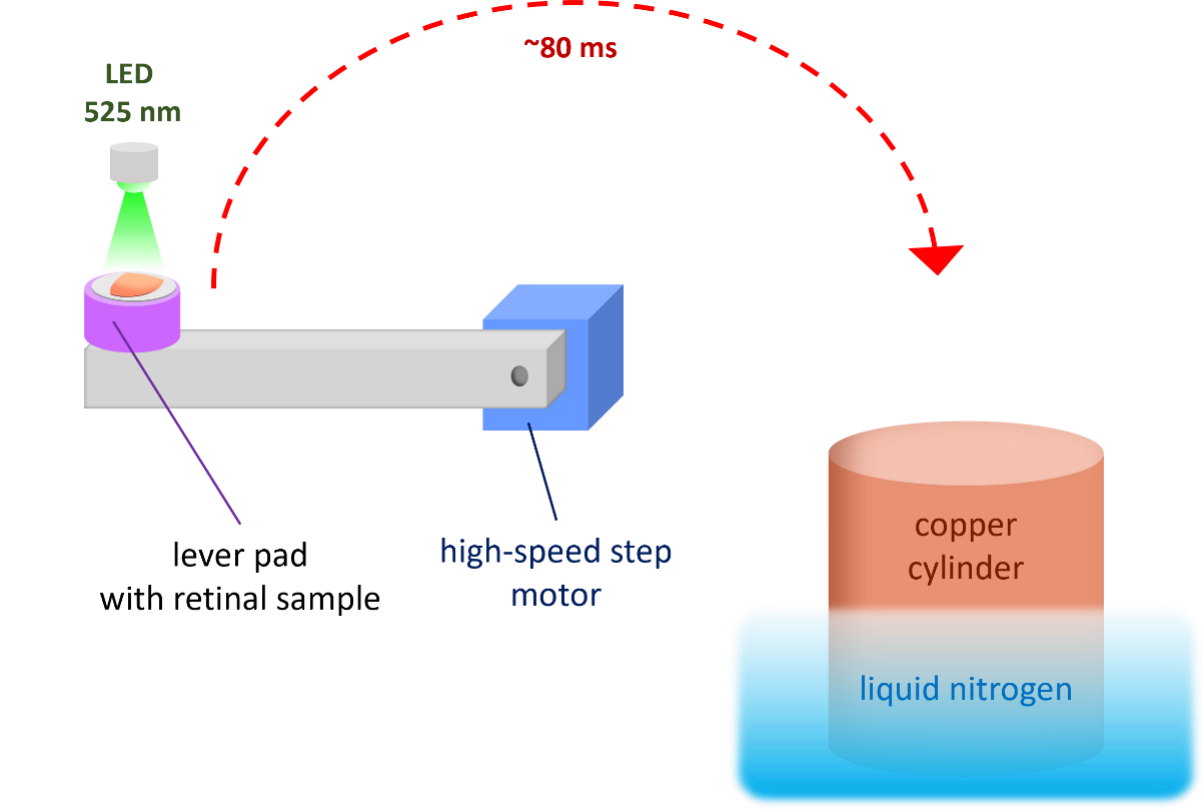
Schematic representation of light stimulation and cryofixation procedure. The retinal sample is placed horizontally on a Tissue-Tek pretreated lever pad. After launching the computer-controlled illumination program (λ_max_ = 525 nm) and following a programmable delay, the lever rotates and presses the sample against a liquid nitrogen–cooled copper cylinder.

1. Pour liquid nitrogen into the container with the copper cylinders to cool them. Maintain a constant level of liquid nitrogen in the container.

2. Pretreat the lever pads with Tissue-Tek.

3. Place the retina specimen horizontally on the pretreated pad to glue it to the lever pads (Figure 2).

4. Start the illumination program with the selected parameters. After a programmable delay, the specimen lever presses the retinal specimen against the polished surface of the copper cylinder (Figure 2).

*Note: After cryofixation, the retinal specimen must be left on the cylinder for a few minutes to allow the Tissue-Tek to freeze properly, making it easier to separate the specimen from the pad with a scalpel.*

5. Separate the cryofixed specimen from the pad with a scalpel.

6. Place the sample in a light-isolated cryo-container with liquid nitrogen and store it for no more than 2 h prior to use on the cryotome.

*Note: After separating the frozen specimen, it is important to allow the pad to warm up to room temperature before applying the next sample with the liquid Tissue-Tek. If the pad remains cold, the specimen may not adhere properly to the pad.*

**C. Slicing of the photoreceptor layer**

1. Preparations

a. Pre-cool the cryotome to -30 °C in advance.

b. Prepare the necessary instruments: tweezers, scalpels, a spatula with thermal insulators, and a hollow metal tube. All instruments must be pre-cooled with liquid nitrogen before each contact with the retinal sample.

c. Create the specimen platform by freezing Tissue-Tek onto a flat circular-patterned chuck.

d. Trim the sample platform by cutting off the rounded edge of the solidified Tissue-Tek to create a flat surface for sample placement.

**Figure 3.**
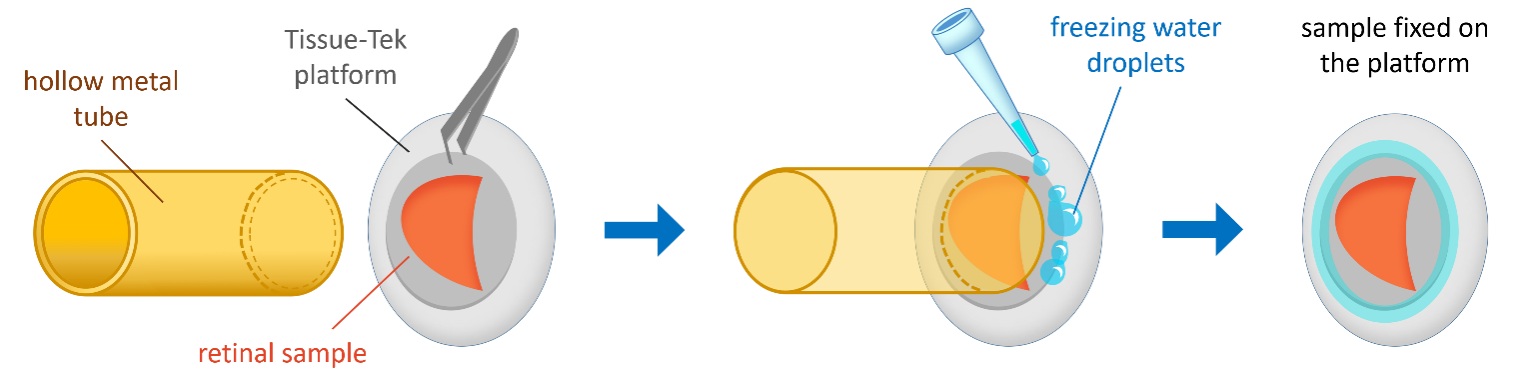
Schematic representation of specimen fixation. The retinal sample is placed on the Tissue-Tek platform with the photoreceptor side facing the knife and pressed down using a hollow metal tube. Small droplets of Milli-Q water are added around the edges of the platform to enhance adhesion by freezing.

2. Specimen fixation

a. Place the specimen on the platform with the photoreceptor side facing the knife and press it down on the platform using the hollow metal tube (Figure 3).

b. Using a pipette with Milli-Q water, add small 10 μL droplets to the edges of the sample to enhance adhesion by freezing. Detach the pressing metal tube (Figure 3).

c. Briefly immerse the sample platform in liquid nitrogen to further cool the sample and freeze the water droplets.

**Figure 4.**
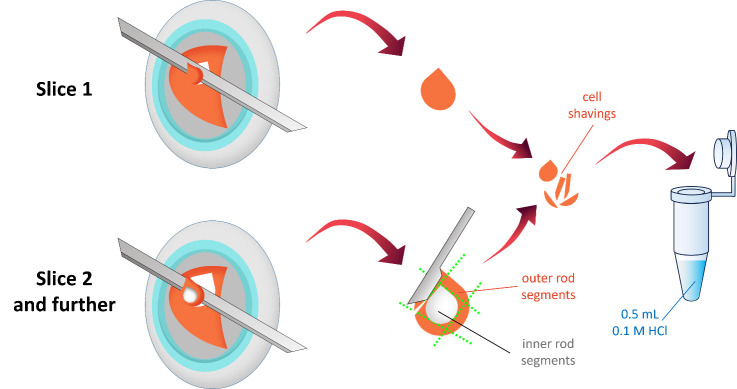
Schematic representation of sample cryotome sectioning. The retina is serially sliced at 10-μm thickness until the orange-pink layer containing outer photoreceptor segments is completely removed. The resulting cell shavings are visually inspected to exclude white inclusion areas, which correspond to the rod inner segment layer. The collected cell shavings are transferred into pre-cooled microcentrifuge tubes containing 500 μL of 0.1 M HCl.

3. Specimen sectioning

a. Serially section the retina longitudinally at 10-μm thickness until the orange-pink layer containing the photoreceptors is completely removed (Figure 4).

b. Visually inspect the resulting cell sections in situ to detect and delete white inclusion areas, corresponding to the rod inner segment layer, from the total material (Figure 4).

c. Collect the cell shavings with a spatula into pre-cooled microcentrifuge tubes containing 500 μL of 0.1 M HCl. The acid is used to inactivate enzymes in the cell shavings.

**D. Sonication and sample preparation**

1. Lyse and disrupt the harvested cell shavings by sonication using a liquid nitrogen–pre-cooled ultrasonic tip.

2. Use the following sonication parameters: 15 s; pulse mode, 3 s on, 1 s off; amplitude 35%; tip diameter 2 mm.

3. Store the resulting suspension at 0.1 °C to avoid freezing until aliquoted. Aliquot 50 μL for protein concentration assay and 450 μL for LC–MS/MS cAMP and cGMP quantification.

4. Lyophilize the aliquot for LC–MS/MS and store at -80 °C.

**E. Protein concentration assay**

*Note: The protein concentration assay must be performed immediately after sample collection, as prolonged exposure to an acidic environment will result in protein denaturation and precipitation, making accurate quantification impossible.*

1. Prepare BSA standards of different concentrations dissolved in 0.1 M HCl, similarly to the samples.

2. Add 200 μL of Bradford reagent to each well of the flat-bottomed low-binding 96-well plate, followed by 15 μL of either the sample solution or the BSA standard. All assays are performed in triplicate.

3. Measure the absorbance at 595 nm using a spectrophotometer.

4. Determine protein concentration by plotting standard curves using Microsoft Excel.

**F. Preparation of standard solutions**

1. Weigh 10 ± 0.1 mg of cAMP-13C5 accurately to prepare the stock internal standard solution.

2. Transfer cAMP-13C5 to a volumetric flask and dissolve in 1,000 mL of 0.1 M HCl water solution. Store internal standard stock solution at -20 °C and bring to room temperature before use.

3. Prepare internal standard solutions of 10 ng/mL by diluting the stock solution with 0.1 M HCl.

4. Weigh 10 ± 0.1 mg of each substance accurately to prepare the standard solutions of cAMP and cGMP.

5. Transfer the weighed samples to different volumetric flasks and dissolve in 1000 mL of the internal standard solution to obtain the corresponding stock solutions.

6. Prepare calibration solutions (0.5, 1.0, 5.0, 10.0, 25.0, 100.0, and 200.0 ng/mL) from the stock solution by diluting in internal standard (IS) solution.

**G. Chromatography sample preparation**

1. Add 50 μL of the internal standard solution to Eppendorf tubes with samples and thoroughly mix using consistent vortexing (15 min, 2,000 rpm, room temperature) and sonicator (15 min, 25 °C, 100 W).

2. Centrifuge the Eppendorf tubes at 13,100× *g* for 5 min after ultrasonic stirring.

3. Transfer approximately 40 μL of the supernatant into glass vials for HPLC analysis.

**H. LC–MS/MS HR technique**

1. Prepare two mobile phases: A = mass-spec solution and B = acetonitrile.

2. Equilibrate the column Zorbax SB-C8 (Column Length × ID × Particle size: 150 mm × 4.6 mm × 1.8 μm, Agilent) with 2% B mixture at a flow rate of 400 μL/min and a temperature of 35 °C.

3. Inject 20.0 μL of sample into the injection port.

4. Start the separation program:

0–2.0 min (2% B)

2.0–8.0 min (2% B to 30% B gradient)

8.0–9.0 min (30% B)

9.0–9.1 min (30% B to 2% B gradient)

9.1–11 min (2% B)

5. Set the MS/MS parameters as follows:

Ionization type: ESI

Ionization polarity: Negative

Electrospray/APCI voltage: 3.5 kV

Scan time: Set by automatic gain control

Mass resolution: 35,000

Type of MS/MS scan: Product ion scan (PIS)

Normalized collision energy: 20%

Collision gas: Nitrogen

Precursor ion(s)/transitions (see Table 1)

**Table 1.** Precursor ion(s)/transitions

Compound	t_R _(min)	MRM1
cAMP	4.87	328.0452 → 134.0457
cGMP	4.81	344.0402 → 150.04109
cAMP-13C5 (IS)	4.87	→ 134,0457

6. Data analysis: The obtained chromatograms were processed in the program ThermoXcalibur Version 4.1.31.9.

7. Integration of analyte peaks was performed automatically using the ThermoXcalibur Qual Browser dialog box with the following parameters:

Mass tolerance: 10.0 ppm

Type of smoothy: Gaussian (number of points, 7)

Minimum of peak height (S/N): 2.0

S/N threshold: 0.5

Peak detection algorithm: Genesis

8. Peaks were identified using the NIST MS Search 2.4 mass spectral library. The identified peaks were verified using chromatograms of standard solutions of the studied substances cAMP, cGMP, and cAMP-13C5 with concentrations of 10 and 100 ng/mL. The identification was performed based on the absolute retention times of the analytes and the ratios of the corresponding characteristic product ions formed during the fragmentation of the precursor ion. The analyte is considered to be identified if:

- The absolute retention time of the analyte does not differ from that established during calibration by more than 0.2 min.

- All characteristic product ions are present with a signal-to-noise ratio (S/N) of at least 3.

- The relative intensities of the analyte product ions (ion ratio, in percentage, relative to the most intense analyte product ion) must correspond to the relative intensities of the standard analyte solution with a concentration closest to the analyte concentration in the analyzed sample. Permissible deviations are given in Table 2.

**Table 2.** Permissible deviation (%) between relative ion intensities for sample and standard solution

Ion ratio	Permissible deviation, %
More than 50	±20
From 20 to 50	±25
From 10 to 20	±30
Less than 10	±50

9. For each analyte peak, the ratio of its area to the peak area of the internal standard cAMP-13C5 was determined. Using standard solutions with certain metrological parameters, a calibration dependence of the analyte concentration on the ratio of its peak area to the peak area of the internal standard was established.

## Validation of protocol

The protein concentration obtained from a single sample typically ranges from 150 to 500 μg/mL, with an average yield of approximately 300 μg/mL. Concentrations below 150 μg/mL are generally considered insufficient for mass spectrometry analysis, as they are unlikely to reach the detection threshold.

The average cAMP content in the dark, quantified by the protocol, was 11.4 ± 0.5 pmol/mg of protein, which transiently elevated after light exposure. The average cGMP level in the dark, quantified by the protocol, was 24.7 ± 0.9 pmol/mg of protein.

This protocol has been used and validated in the following research article:

• Chernyshkova et al. [13]. Light induces a rapid increase in cAMP and activates PKA in rod outer segments of the frog retina. *Journal of General Physiology*.

## General notes and troubleshooting

The retina of all vertebrates has a very similar structure; therefore, the cryofixation and isolation approach for samples of the outer segment layer, as described in this article, is quite universal and can be applied to any retina. However, it is important to consider the significant differences in the linear dimensions of the eye and, consequently, the retina, as well as the thickness of the cellular layers of the retina, particularly the photoreceptor layer. For example, the diameter of a frog's eye (*Rana ridibunda*) is 6–8 mm [14], whereas a mouse's eye is 3.2–3.5 mm [15], making the mouse's retinal area 4 times smaller. In addition, the outer segments of mouse rods are 1.5 times shorter than those of frogs (24 μm vs. 35 μm; our own measurements) [16]. Consequently, the total photoreceptor layer sample volume that can be obtained from a single mouse eye is approximately eight times smaller than that from a single frog eye, making it challenging to apply this technique to small rodent eyes. One possible solution would be to use the entire retina of one eye for dark control and the entire retina of the second eye after light illumination.

The viability and robustness of the mammalian retina compared to the frog retina may pose another challenge. Generally, specimens of mammalian retina need more effort to preserve their viability during the experiment. For instance, they may require thermal stabilization corresponding to the animal body temperature, oxygenation, or specific additives to the dissection solutions.

**Troubleshooting**

**Problem 1:** Initially, the rapid movement of the stepper motor lever caused the sample to fly off the lever pad before it could reach the cooled surface of the copper cylinder (the slingshot effect). Initially, we tried gluing the samples to the arm pad using Tissue-Tek gel at room temperature. However, this resulted in another problem: the gel froze with the sample, making it very difficult to separate the sample from the arm pad.

**Solution:** We redesigned the lever pad so that a fluoroplastic insert occupied most of its surface area. We found experimentally that the frozen Tissue-Tek gel could easily be separated from the fluoroplastic with a scalpel.

**Problem 2:** To improve the extraction of cAMP from outer segment fragments dissolved in 0.1 M HCl, we used a sonicator (see Materials). When using a sonicator, the temperature of the sonicator rod must be carefully controlled because a rod that is too warm can heat the photoreceptor fragment suspension above the standard temperature, while a rod that is too cold can freeze the sample and collect some of the suspension on itself.

**Solution:** We cooled the sonicator rod by immersing it in liquid nitrogen for a strictly controlled time, chosen in advance through experimentation. This time should obviously be selected individually for each sonicator model.

**Problem 3:** In order to investigate the dynamics of changes in signaling metabolites in frog photoreceptors, we employed low levels of physiological light stimulation. Under these conditions, it is particularly important to isolate the retinal sample from any light contamination in order to ensure the reproducibility of the experiment.

**Solution:** We performed all experiments in a completely darkened room. After placing the retinal specimens on the arm pads, however, we covered the entire setup with a light-tight cover, switched off the control computer monitors, and started cryofixation using a dedicated hardwire button.
